# Target localization accuracy in frame‐based stereotactic radiosurgery: Comparison between MR‐only and MR/CT co‐registration approaches

**DOI:** 10.1002/acm2.13580

**Published:** 2022-03-14

**Authors:** Eleftherios P. Pappas, Ioannis Seimenis, Panagiotis Kouris, Stefanos Theocharis, Kostas I. Lampropoulos, Georgios Kollias, Pantelis Karaiskos

**Affiliations:** ^1^ Medical Physics Laboratory Medical School National and Kapodistrian University of Athens Athens Greece; ^2^ Medical Physics and Gamma Knife Department Hygeia Hospital Marousi Greece

**Keywords:** distortion, frame, Gamma Knife, registration, spatial accuracy, SRS, stereotactic space

## Abstract

**Purpose:**

In frame‐based Gamma Knife (GK) stereotactic radiosurgery two treatment planning workflows are commonly employed; one based solely on magnetic resonance (MR) images and the other based on magnetic resonance/computed tomography (MR/CT) co‐registered images. In both workflows, target localization accuracy (TLA) can be deteriorated due to MR‐related geometric distortions and/or MR/CT co‐registration uncertainties. In this study, the overall TLA following both clinical workflows is evaluated for cases of multiple brain metastases.

**Methods:**

A polymer gel‐filled head phantom, having the Leksell stereotactic headframe attached, was CT‐imaged and irradiated by a GK Perfexion unit. A total of 26 4‐mm shots were delivered at 26 locations directly defined in the Leksell stereotactic space (LSS), inducing adequate contrast in corresponding T2‐weighted (T2w) MR images. Prescribed shot coordinates served as reference locations. An additional MR scan was acquired to implement the “mean image” distortion correction technique. The TLA for each workflow was assessed by comparing the radiation‐induced target locations, identified in MR images, with corresponding reference locations. Using T1w MR and CT images of 15 patients (totaling 81 lesions), TLA in clinical cases was similarly assessed, considering MR‐corrected data as reference. For the MR/CT workflow, both global and region of interest (ROI)‐based MR/CT registration approaches were studied.

**Results:**

In phantom measurements, the MR‐corrected workflow demonstrated unsurpassed TLA (median offset of 0.2 mm) which deteriorated for MR‐only and MR/CT workflows (median offsets of 0.8 and 0.6 mm, respectively). In real‐patient cases, the MR‐only workflow resulted in offsets that exhibit a significant positive correlation with the distance from the MR isocenter, reaching 1.1 mm (median 0.6 mm). Comparable results were obtained for the MR/CT‐global workflow, although a maximum offset of 1.4 mm was detected. TLA was improved with the MR/CT‐ROI workflow resulting in median/maximum offsets of 0.4 mm/1.1 mm.

**Conclusions:**

Subpixel TLA is achievable in all workflows. For the MR/CT workflow, a ROI‐based MR/CT co‐registration approach could considerably increase TLA and should be preferred instead of a global registration.

## 1 INTRODUCTION

Stereotactic radiosurgery (SRS) is an advanced radiotherapy approach commonly used for the treatment of multiple brain metastases.^1^, ^2^ Its main characteristic is that high doses (typically ∼20–24 Gy) are delivered to the targets either in a single or a limited number of sessions. Thus, spatial accuracy is of paramount importance, especially if millimeter‐level lesions are involved. A geometric offset of the order of 1 mm or 1° can induce a considerable dosimetric impact on both target and adjacent critical organs.^3^, ^4^, ^5^, ^6^, ^7^, ^8^, ^9^ Application of margins around the identified lesions (determining the planning target volume, PTV) may warrant target dose coverage, at the expense of a potentially increased risk for radiation‐induced brain necrosis.^10^, ^11^, ^12^, ^13^, ^14^ Therefore, it is crucial that applied margins are relevant to the overall spatial uncertainties of the workflow followed but should not be unnecessarily large.

Frame‐based Gamma Knife (GK) (ELEKTA AB, Stockholm, Sweden) SRS is a well‐established treatment modality for multiple brain metastases. Briefly, the Leksell stereotactic headframe is pinned to the patient's head (i) for immobilization during imaging and treatment delivery and (ii) to spatially register the coordinate system of the primary image volume (either computed tomography [CT] or magnetic resonance [MR] images) to the coordinate system of the Leksell stereotactic space (hereinafter to be referred to as LSS, used for treatment delivery). The latter is achieved by acquiring images after fixing the Leksell localization box on the stereotactic headframe. The imaged N‐shaped fiducials are identified by the dedicated ELEKTA GammaPlan (ELEKTA AB, Stockholm, Sweden) treatment planning system (TPS) on the primary image stack and an automatic least‐square fitting process (with the mechanically expected shape and size of the fiducials) is performed to determine the most optimum rigid transformation, linking the imaging and treatment coordinate systems.^15^ Accuracy of this spatial registration step is essential for accurate dose delivery.

Depending on the image modality used as the primary volume for treatment planning, two frame‐based workflows are clinically implemented^15^; (i) treatment planning is performed directly on the MR images involving the headframe and the MR‐compatible localization box (hereinafter, the “MR‐only” workflow) and (ii) the “MR/CT” workflow where the CT images contain the (CT‐compatible) N‐shaped fiducials and MR images are employed to assist in target delineation through an anatomy‐based co‐registration step with the CT images (which are already registered to the LSS). Thus, the latter approach involves two spatial co‐registration steps (linking the MR/CT/LSS coordinate systems) while the former involves only one registration step (linking the MR/LSS coordinate systems).

Nevertheless, contrast‐enhanced T1‐weighted (T1w) MR images are always used in order to take advantage of the superior soft tissue contrast between the lesions (owing to the contrast agent uptake) and the surrounding normal brain parenchyma. However, MR images inherently exhibit geometric distortion which may affect target localization accuracy (TLA).^16^ The MR‐related spatial distortions are conveniently grouped into two categories. Distortions stemming from main magnetic field (B_0_) inhomogeneity, tissue susceptibility differences, and the chemical shift effect are collectively referred to as sequence‐dependent distortions, also involving patient‐induced distortion.^17^, ^18^ Their common characteristic is that distortion sign changes upon read gradient polarity reversal (e.g., the frequency encoding axis is reversed from anterior‐posterior to posterior‐anterior) in conventional three‐dimensional (3D) non‐echo planar (EP) images, while distortion magnitude remains unaffected. This has been exploited in distortion detection and/or correction studies.^5^, ^18^, ^19^, ^20^, ^21^, ^22^ For a field of view relative to a cranial MR scan, an average of 0.5 mm of sequence‐dependent distortion is expected.^23^, ^24^ On the other hand, nonlinearity of the gradient fields are only system‐related and are known as sequence‐independent distortions.^17^, ^18^ Since sequence‐independent spatial distortion does not relate to the patient being scanned, scanner manufacturers have developed and implemented postimaging distortion correction routines that minimize gradient nonlinearity‐related effects, especially in the limited field of views used in cranial scans.^25^, ^26^ Either sequence‐dependent or sequence‐independent, distortion generally increases with increasing distance from the MR isocenter and might compromise target localization and delineation.

The inherent uncertainty of the anatomy‐based MR/CT spatial co‐registration procedure should also be accounted to the overall uncertainty budget in MR/CT workflows. According to a multi‐institutional benchmark study, an uncertainty of 1.8 mm should be considered for cranial SRS cases.^27^ In another study employing specifically the GammaPlan TPS, mean registration errors of up to 1 mm were reported.^28^ Nevertheless, accuracy of this step is also user‐dependent. The selected volume of interest, defining the anatomy to be taken into account by the corresponding optimization algorithm as well as the initial relative positions of images may also affect registration accuracy.^29^, ^30^, ^31^

This study aims at determining the overall TLA in SRS procedures, using the MR‐only workflow utilized in GK frame‐based applications, as well as when the MR/CT approach is followed to co‐register MR with corresponding CT images (with the latter used to define the LSS coordinate system) based on patient anatomy. For the MR‐only workflow, this is affected by MR distortion at the target location and at the N‐shaped fiducials. For an MR/CT workflow, the extra (anatomy‐based) MR/CT co‐registration step along with MR distortion at the target location and surrounding anatomical landmarks contribute to the target localization uncertainty budget. The scope of this work has been served by conducting both a phantom and a patient study. Regarding the former, a container filled with polymer gel was irradiated in order to introduce MR contrast at coordinates, directly defined in the LSS, serving as the reference for targeting accuracy assessment. In addition to the two clinical workflows, distortion‐corrected MR images have also been evaluated (hereinafter, “MR‐corrected” workflow) in order to determine their suitability to serve as the reference for the patient study. To evaluate workflows employing the clinical pulse sequences and imaging parameters, as well as MR/CT co‐registration procedures of realistic contrast, distortion, and noise levels, the two clinical workflows were also evaluated using patient images, acquired for GK frame‐based SRS of multiple brain metastases. Both global and region of interest (ROI)‐based^29^, ^30^, ^31^ MR/CT co‐registration approaches were considered for the evaluation of the MR/CT workflow.

## 2 METHODS

### 2.1 Phantom study

#### 2.1.1 Phantom preparation

A phantom presented in a previous study^23^ has been utilized. Briefly, a hollow spherical container made of acrylic with a diameter of 16 cm that is similar to the size of a human head and, therefore, compatible with the Leksell stereotactic headframe model G (ELEKTA AB, Stockholm, Sweden) and accompanying localization boxes, has been used during image acquisitions. The container was filled with a normoxic polymer gel dosimeter referred to as VIP.^32^, ^33^ This formulation is characterized by tissue‐equivalent radiological properties and exhibits linear dose–response behavior up to at least 30 Gy.^33^ Details on the gel's chemical composition and dose–response characterization can be found in the relevant literature. The protocol followed for preparation, storage, and handling has been described in a previous publication.^23^

Upon irradiation, monomer components of the gel are polymerized, resulting in a considerable reduction of the T2 relaxation time.^34^ This characteristic is exploited for gel dose read‐out using a clinical MRI system and T2‐weigthed (T2w) pulse sequences.^34^ However, for the purposes of the present study, it should be noted that the gel was not utilized as a dosimeter, that is, a dose distribution was not derived from the gel read‐out. Radiation‐induced polymerization offers the necessary contrast in T2w MR images, serving as hypothetical targets. The key advantage of this approach is that, following irradiation, the targets/contrast can be determined and their positions can be compared with the nominal ones, defined directly in the LSS coordinate system.

#### 2.1.2 Treatment delivery, image acquisitions, and treatment planning

A GK Perfexion unit (ELEKTA AB, Stockholm, Sweden) was used for treatment delivery. The phantom, with the stereotactic headframe and the appropriate localization box, underwent a CT scan for GK treatment planning purposes by employing a SIEMENS Somatom Definition scanner (SIEMENS Healthineers, Enlargen, Germany). Reconstructed voxel size was 0.43 × 0.43 × 1 mm^3^.

A total of 26 GK shots were delivered to the gel, with all eight sectors always aligned with the 4 mm collimator openings. Using the dedicated TPS (GammaPlan v.11.1.1, ELEKTA AB, Stockholm, Sweden), the shots were prescribed in the LSS at coordinates selected to cover the entire useful volume of the phantom. One shot was positioned at the unity center point (UCP, supposed to coincide with the radiation focus point [RFP]; the point in space where the central axes of all sources intersect^15^) with nominal LSS coordinates of (*x, y, z*) = (100,100,100) mm. The remaining 25 shots were symmetrically distributed toward all three normal axes, extending up to the phantom edges. Prescription dose for each shot was 12.5 Gy delivered to the 50% isoline.

**FIGURE 1:**
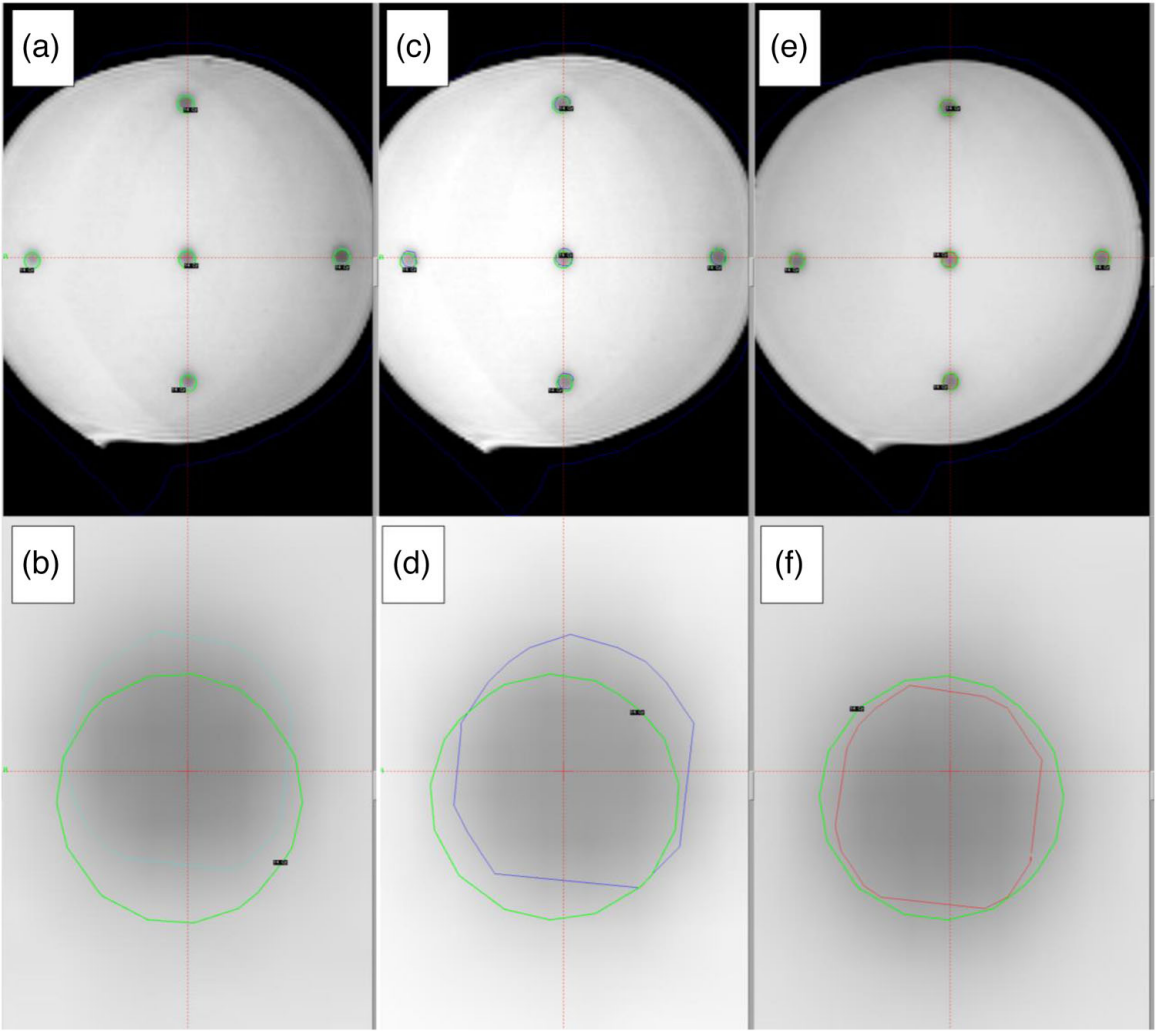
Screenshots of the GammaPlan treatment planning system depicting a transversal magnetic resonance (MR) slice of the irradiated phantom intersecting five Gamma Knife (GK) shots, after performing the spatial co‐registration to the Leksell stereotactic space (LSS), following the (A,B) MR‐only, (C,D) MR/CT, and (E,F) MR‐corrected workflows. To increase visibility, the central GK shot is enlarged in the bottom row panels (B,D,F). Legend: Green solid line: the 14‐Gy isodose for each GK shot delivered (corresponding centroids serving as reference points); light blue, dark blue, and red lines: contours of the automatically generated target structures, defined using the radiation‐induced polymerization areas, for the assessment of target localization accuracy, following the MR‐only, MC/CT, and MR‐corrected workflows, respectively. All data shown are registered to the LSS coordinate system

Subsequently, the irradiated phantom (with the stereotactic headframe and MR localization box) was MR‐scanned, using a Philips Intera 1.5T unit (Philips Medical Systems, The Netherlands) and the dedicated frame‐compatible head coil. More specifically, 3D T2w turbo spin echo (TSE) images were acquired at a voxel size of 1 × 1 × 1 mm^3^, while the anterior‐posterior direction was selected for frequency encoding (series #1, Table 1). All available vendor‐supplied distortion correction algorithms were enabled for all sequences employed and all implemented workflows.^25^, ^26^ An indicative transversal MR slice intersecting with five shots is shown in Figure 1. Although these imaging parameters are not clinically used in GK treatment planning for multiple brain metastases (where contrast‐enhanced T1w MR images are employed to define the targets), they provide optimum contrast between high‐ and low‐dose areas of the irradiated phantom. Moreover, corresponding images exhibit typical levels of both sequence‐dependent and sequence‐independent MR‐related spatial distortion.^23^ Thus, radiation‐induced polymerization area can be used to define each target and TLA of the workflow used can be defined as the offset between the polymerization area centroid and the corresponding shot centroid.

**TABLE 1:** Magnetic resonance (MR) pulse sequences and imaging parameters used for the phantom and patient studies (image series #1‐2 and #3‐4, respectively)

#	MR pulse sequence	Pixel bandwidth (Hz/px)	Imaging parameters TE/TR/FA/ETL (msec/msec/o/‐)	Read gradient axis and polarity	Acquisition time per 100 slices (min)	Voxel size (mm^3^)
1	T2w 3D Turbo Spin Echo	146.2	160/2700/90/50	A‐P (*y*‐axis)	13.5	1 × 1 × 1
2	T2w 3D Turbo Spin Echo	146.2	160/2700/90/50	P‐A (*y*‐axis)	13.5	1 × 1 × 1
3	Contrast enhanced T1w 3D spoiled GRE	156.8	2.17/25/30/‐	A‐P (*y*‐axis)	9.4	0.82 × 0.82 × 1.0
4	Contrast enhanced T1w 3D spoiled GRE	156.8	2.17/25/30/‐	P‐A (*y*‐axis)	9.4	0.82 × 0.82 × 1.0

Abbreviations: A, anterior; ETL, echo train length; FA, flip angle; GRE, gradient recalled echo; P, posterior; TE, echo time; TR, repetition time.

In order to implement the mean image distortion correction technique^5^ (see subsection MR‐corrected workflow in Section 2.1.3 for details), an additional MR scan was performed using identical imaging parameters, except for the reversal of read gradient polarity, that is, the posterior‐anterior direction was used for frequency encoding (series #2, Table 1). As a result, magnitude of MR‐related distortions will not be affected but sequence‐dependent distortion (also phantom‐ or patient‐induced) will change sign.^17^, ^18^, ^19^ This is exploited in MR distortion correction (subsections MR‐corrected workflow and Reference dataset in Sections 2.1.3 and 2.2.3, respectively).

#### 2.1.3 Workflows evaluation

##### Reference dataset

For identification purposes, all delivered shots were numbered sequentially starting from the one positioned at the UCP/RFP. The centers of the shots were defined in the LSS and their coordinates were verified in the treatment report, constituting a set of 26 well‐defined points, within the GK treatment space that were used as the reference dataset. It should be noted that the CT images were only used to choose the most suitable positions of shots in order to cover the entire useful volume of the gel. The nominal shot centers were regarded as the reference dataset for the evaluation of all workflows’ TLA, provided that the stereotactic headframe had not moved between treatment delivery and image acquisitions and the mechanical uncertainties of the robotic couch and RFP/UCP coincidence are minimal (measured at 0.1 mm, see Section 4). Indicatively, for five GK shots, reference locations represented by the centroids of the isolines are shown in Figure 1.

##### MR‐only workflow

Acquired MR images were imported to the TPS for treatment planning. The N‐shaped fiducials of the MR localization box was used for automatic registration to the LSS coordinate system, leading to a mean definition error of 0.3 mm (mean and maximum fiducial position error in image planes 0.3 and 0.9 mm, respectively). Subsequently, polymerized volumes of the phantom were reviewed and examined for inconsistencies, deficits, or insufficient image quality. All 26 shots were successfully identified and were assigned the number corresponding to the reference ones (i.e., pairing reference and evaluated datasets). Each shot was regarded as an independent lesion. Target contouring was performed by employing an automatic approach based on thresholding of the pixel intensities, exploiting the MR contrast between high‐ and low‐dose areas. Obtained contours were also reviewed and verified by an experienced planner. The validation criteria involved checking for symmetry and round shape, as expected from the dose shape of the GK 4 mm shots. Five indicative structures resulting from this process are shown in Figure 1A,B.

The final treatment plan was exported from the TPS in DICOM‐RT format as well as in an xml file. The latter contains the rigid transformation matrix, which was automatically calculated by the TPS and used to spatially register the imported MR images to the coordinate system of the LSS (i.e., the MR/LSS rigid transformation matrix). The former contains the coordinates of all vertices related to all contours created, registered to the DICOM coordinate system of the MR images.

##### MR/CT workflow

In order to simulate the clinical MR/CT workflow, CT images of the phantom with the CT‐compatible localization box were imported to the TPS. In order for the CT volume to serve as the primary image stack for treatment planning, the N‐shaped fiducials were identified and the CT/LSS spatial registration was performed. MR images were also imported and spatially co‐registered to the CT images by using the mutual information algorithm, implemented in the TPS. More specifically, a rigid transformation matrix was calculated based on anatomical information of the two image stacks. This calculation relied on the entire volume of the phantom (i.e., a global registration approach), but the N‐shaped fiducials and the headframe were not included.

Shot identification and target contouring were performed in the registered MR images, as described above. Since the same MR images and methodology were employed for target contouring, same contour shapes were obtained; however, these contours were not necessarily lying at the same locations within the LSS (Figure 1C,D). An experienced planner reviewed and verified all 26 automatically generated targets.

Subsequently, all created structures were exported in DICOM‐RT format. Moreover, both rigid transformation matrices (corresponding to the MR/CT and CT/LSS spatial registrations) calculated by the TPS were exported in the relevant xml file.

##### MR‐corrected workflow

The mean image distortion correction technique has been proposed in the work of Karaiskos et al.^5^ and evaluated in subsequent studies involving both phantom and patient studies.^21^, ^22^ Briefly, two MR scans were performed with opposing read gradient polarities. The two images (considered as a priori spatially co‐registered, provided that the patient did not move) were combined into one, by averaging the corresponding pixel intensities on a pixel‐by‐pixel basis, which is literally the mean image. This simple‐in‐concept distortion correction technique can effectively minimize sequence‐dependent distortions, which are also patient‐induced.^22^ It should be noted that images used for this workflow were already corrected for gradient nonlinearity distortion, after having enabled the relevant algorithm provided by the vendor.

For the purposes of the present study, image series #1,2 (Table 1) were used to implement this technique. All relevant MR images in DICOM format were imported to MATLAB R2019a (The MathWorks, Inc., Natick, MA). A custom in‐house routine was developed to read, average the intensities of the two image stacks, and export the created image series in DICOM format.

It should be noted that signal averaging is performed for the entire image volume, including the positions of the N‐shaped fiducials which are also affected. Moreover, this processing step does not affect any of the image parameters, such as the pixel size or slice thickness. Image contrast is slightly enhanced compared to the original images.^22^

Distortion‐corrected images were imported to the TPS for treatment planning. The MR‐only approach was followed, that is, no CT images were used. Targets were contoured by implementing the same methodology as described above for the MR‐only workflow (five are shown in Figure 1E,F). All created data and calculated rigid transformations were exported in DICOM‐RT and xml file formats, respectively.

#### 2.1.4 Analysis and comparison

In order to compare reference shot locations with the ones identified by following each of the three workflows considered in the phantom study, structures and xml files were imported to MATLAB. Custom routines were developed to read vertex coordinates for each contour, which were registered to the MR coordinate system, irrespective of the workflow followed. Using the rigid transformation matrices (calculated by the TPS and written in the xml file), vertex coordinates were transformed to LSS. More specifically, in the case of the MR‐only and MR‐corrected workflows, the respective MR/LSS transformations were applied, while for the MR/CT workflow, two consecutive rigid transformations were performed (i.e., MR/CT and CT/LSS).

After determining the vertex coordinates of all contours in the stereotactic space, the next step involved calculation of the structure's centroid. For this purpose, a grid of voxels (at a resolution of 0.1 × 0.1 × 0.1 mm^3^) was created, bounding the entire structure in 3D. For each voxel, using Delaunay triangulation and standard embedded MATLAB routines, it was determined whether the center of each voxel lies within the outline of the contour or on the outside. This approach allowed for the creation of a binary matrix with voxels of 0.001 mm^3^ enclosed by the structure assigned the value of 1. Centroid calculation was followed using standard MATLAB routines for binary matrices. This contour processing step was mainly adapted from the one presented in the work of Growcott et al.^35^

### 2.2 Patient study

#### 2.2.1 Patient selection

Selection criteria among the cases of multiple brain metastases considered for inclusion in the patient study were (i) target size limited to 3cc, (ii) lesions that do not include necrotic cores, (iii) well‐defined lesion borders exhibiting contrast with the surrounding normal brain parenchyma, and (iv) targets that are not adjacent to a vessel or other structures yielding increased T1w MR signal. The above criteria warrant the applicability of the evaluation method employed and, more specifically, ensure that the thresholding step for automatic contouring does not fail and can also provide almost identical target volumes when applied to the MR distortion‐corrected images. Moreover, effort was made to include cases with several lesions lying distant from the MR isocenter and distant from one another as being more prone to distortions and potentially suboptimal MR/CT spatial registration. A total of 15 patients with 81 brain metastases were enrolled in this study, approved by the Institutional Ethics Committee. Details related to the physical characteristics of the lesions are summarized in Table 2.

**TABLE 2:** Physical characteristics of the 81 lesions (15 patients) included in the patient study

Lesion characteristic	Minimum	Maximum	Median
Lesions per patient	2	10	5
Volume (mm^3^)	1.4	2833.2	92.4
Distance to MR isocenter (mm)	19.1	105.6	62.3
Distance to UCP/RFP (mm)	15.8	92.1	59.6

Abbreviations: MR, magnetic resonance; RFP, radiation focus point; UCP, unity center point.

#### 2.2.2 Image acquisitions

Each patient underwent the clinical MR/CT workflow for GK frame‐based SRS. Briefly, the Leksell stereotactic headframe (along with the CT‐compatible localization box) was pinned on the head of the patient and a CT scan was performed at 120 kVp using a SIEMENS Somatom Definition scanner. No contrast agent was administered at this step for the vast majority of the patients (12/15). Subsequently, the MR‐compatible localization box was mounted to the headframe and MR images were acquired using the dedicated frame‐compatible head coil and a Philips Intera 1.5T scanner, following an intravenous injection of 0.2 mmol/kg gadolinium diethylenetriaminepentaacetic acid (Gd‐DTPA). Scanning parameters are given in Table 1 (series #3). It should be noted that 3D distortion correction algorithms provided by the vendor were enabled for all images acquired and all workflows considered. These algorithms minimize gradient nonlinearity‐related distortion.^25^, ^26^

In accordance with the phantom study, an additional MR scan was performed with identical imaging parameters, except for a reversal of the read gradient polarity (series #4, Table 1). This sequence allowed for the implementation of the mean image correction method that has been shown to minimize sequence‐dependent MR distortions (i.e., stemming from B_0_ inhomogeneity, susceptibility differences, and the chemical shift effect), which are also patient‐induced.^21^, ^22^

#### 2.2.3 Workflows evaluation

##### Reference dataset

According to the phantom study results (see Section 3.2), the MR‐corrected workflow demonstrated excellent TLA with median centroid offset being similar to the combined uncertainty involved, stemming from the centroid localization algorithm and the mechanical dose delivery uncertainties (see Sections 3.1, 3.2, and 4). In addition, the accuracy of this distortion correction approach has been verified in previous works, employing specifically T1w sequences used herein.^21^, ^22^ Moreover, distortion correction is applied to the entire image volume, affecting the position of the N‐shaped fiducials as well, which are used by the TPS to determine the MR/LSS rigid transformation. This correction is additional to the vendor‐supplied postimaging distortion correction process that accounts for gradient nonlinearities. Thus, for the purposes of the patient study, corresponding target centroids defined in the LSS using the MR‐corrected workflow were regarded as the reference dataset for evaluating the TLA of the clinical MR‐only, MR/CT‐global, and MR/CT‐ROI workflows. Target contouring was performed on the corrected MR image series (created from image series #3,4, Table 1), following the MR‐only approach. For all patients and lesions considered, the reference centroid locations were determined by implementing the methodology and relevant routines described in subsection MR‐corrected workflow in Section 2.1.3 and Section 2.1.4.

##### MR‐only workflow

The clinical MR‐only workflow relies solely on images of series #3 (Table 1) with vendor‐supplied MR‐image distortion correction enabled in accordance with the phantom study. The respective phantom study methodology (as described in subsection MR‐only workflow, Section 2.1.3) was also employed here. Similar to the phantom study, the N‐shaped fiducials of the MR localization box were used for automatic registration to the LSS coordinate system, leading to a mean definition error of 0.3 mm (mean and maximum fiducial position error in image planes 0.3 and 1 mm, respectively). Regarding the threshold levels used for automatic target contouring, they slightly varied according to the local signal intensity of the pixels and contrast agent uptake in each lesion. Target volume centers were not sensitive to small variations of threshold levels. Created structures and the relevant MR/LSS rigid transformation matrix were exported in DICOM‐RT and xml file formats, respectively.

##### MR/CT‐global and MR/CT‐ROI workflows

According to the clinical MR/CT workflow, both CT and MR images (series #3, Table 1) were used for treatment planning. However, only the CT images were registered to the LSS using the N‐shaped fiducials and MR images were registered to the LSS through spatial co‐registration with the registered CT images.

Regarding the MR/CT spatial co‐registration, the mutual information algorithm (incorporated in the TPS) was used to derive the most optimum rigid transformation matrix, based on the patient's anatomical information included in a rectangular volume of interest selected by the user. After performing an initial manual registration, two different approaches were considered for this step; (i) the volume of interest included the patient's entire anatomy image except for the neck and cervical spine regions that are known to be affected by MR distortion induced by the presence and proximity to the frame base.^36^ Hereinafter, this approach will be referred to as “MR/CT‐global” workflow and can be regarded as a global image registration methodology. (ii) The rectangular volume of interest included only the central brain area and nasopharyngeal and optic pathway regions, and a ROI‐based automatic registration was performed. Re‐registration was attempted wherever necessary. The latter strategy ensures that the registration relies on the central MR image volume, avoiding areas distant from the MR isocenter where distortions considerably increase. For the purposes of the present study, a workflow involving an MR/CT image registration step of such approach will be referred to as “MR/CT‐ROI” workflow.

Either in the MR/CT‐global or MR/CT‐ROI workflow, all fused MR/CT images were reviewed, and spatial co‐registration was adjusted if necessary. The recommendations^29^ of AAPM TG‐132 for SRS applications were followed (which correspond to level 0 of the registration uncertainty assessment scale), in which an alignment within 1 mm was achieved after checking for coincidence between several random distinct anatomical landmarks identified in the two images.

Since lesion identification and delineation relied solely on MR images, the same threshold values were used for automatic contouring, as the ones selected for the MR‐only workflow. MR/CT and CT/LSS rigid transformation matrices, as well as the created structures, were exported from the TPS for further analysis.

#### 2.2.4 Analysis and comparison

All contours and transformation matrices were imported and analyzed in MATLAB using the same routines as the ones described in Section 2.1.4 for the phantom study. For all patients and workflows, each lesion was processed independently, each resulting to an independent assessment of the corresponding overall TLA. Structure vertices were transformed to the LSS and corresponding centroid locations were calculated. Results related to the MR‐corrected workflow served as the reference dataset (see Reference dataset) for the evaluation of the clinical (MR‐only, MR/CT‐global, and MR/CT‐ROI) workflows. Offsets between reference and evaluated points provide an estimate of the overall TLA related to the workflow considered.

### 2.3 Methodology validation and uncertainty estimation

To serve the goals of this study, a series of in‐house MATLAB routines were developed for (i) reading DICOM‐RT and xml files exported from the TPS, (ii) rendering structures in 3D from vertices, (iii) applying rigid transformation(s), and (iv) calculating the 3D coordinates of the centroid of a transformed structure. In order to validate the developed routines, as well as estimate the uncertainty of the centroid calculation algorithm, a reference set of data was created. In specific, on the MR and CT images of one patient, a set of three cylindrical arbitrary targets were carefully created using the TPS. The arbitrary targets were positioned at well‐defined coordinates in the LSS. This was ensured by prescribing GK shots at the given LSS coordinates and verifying that the corresponding isolines are concentric with the created structures. The same approach was followed for both the MR‐only and the MR/CT workflows. DICOM‐RT and xml files were exported, while the GK treatment report included the reference GK shot centers, directly prescribed in the LSS coordinates. All data were imported to MATLAB; the methodology and routines described above were implemented and obtained centroids in the LSS coordinate system were compared with the reference locations, following both workflows.

## 3 RESULTS

### 3.1 Methodology validation and uncertainty estimation

In all workflows and hypothetical lesions considered for the validation and uncertainty estimation test (see Section 2.3), maximum deviation between calculated and reference locations was 0.16 mm (radial distance). This value has been adopted as an estimate of the uncertainty of the centroid calculation algorithm (worst case scenario). The overall uncertainty budget for both phantom and patient studies is discussed in Section 4. Moreover, this check can be regarded as a validation procedure for the developed structure processing steps, and particularly the application of the rigid transformations between coordinate systems, extracted from the xml files.

### 3.2 Phantom study

In Figure 1, phantom study reference locations (shot centers) are represented by the centers of the round isolines. Detected targets are contoured following all three workflows. For the clinical workflows, spatial offsets from reference locations are evident (Figure 1A‐D). The MR‐corrected workflow demonstrates minimum spatial offset, not visually detectable in Figure 1E,F.

Results related to all 26 shots are summarized in Table 3. The MR‐corrected workflow performs best with a median spatial offset of 0.22 mm, while the maximum detected offset is limited to 0.45 mm. Corresponding values for the clinical MR‐only workflow are 0.82 and 1.24 mm, respectively (Table 3). The main component is found on the *y*‐axis (posterior‐anterior direction, affected by sequence‐dependent distortion) and the *z*‐axis (inferior‐superior direction, affected by the distorted position of the N‐shaped fiducials in *y*‐axis due to sequence‐dependent distortion which also affects localization accuracy in *z*‐axis). TLA considerably improves in the MR/CT workflow, where a CT scan is used for spatial registration to the LSS. However, values presented in Table 3 do not represent a realistic clinical case, where MR/CT registration might be more challenging while patient‐induced MR distortion might also affect the overall accuracy.

**TABLE 3:** Summary of the phantom study results. Median and maximum offset between reference and evaluated centroids in the Leksell stereotactic space (LSS), related to all 26 Gamma Knife (GK) shots and three workflows

	Median absolute offset (mm)				Maximum absolute offset (mm)			
	Δ*x*	Δ*y*	Δ*z*	*R*	Δ*x*	Δ*y*	Δ*z*	*R*
MR‐only	0.0	0.7	0.4	0.8	0.1	1.1	0.8	1.2
MR/CT	0.2	0.4	0.1	0.6	0.7	0.9	0.4	0.9
MR‐corrected	0.1	0.1	0.0	0.2	0.4	0.4	0.2	0.5

*Note*: *x, y, z* correspond to the normal axes of the Leksell stereotactic space (LSS); *x*: left‐right direction; *y*: posterior‐anterior direction; *z*: inferior‐superior direction; *R*: the radial distance between reference and evaluated points, i.e., $R=\sqrt{\Delta x^{2}+\Delta y^{2}+\Delta z^{2}}$.

Abbreviations: CT, computed tomography; MR, magnetic resonance.

### 3.3 Patient study

**FIGURE 2:**
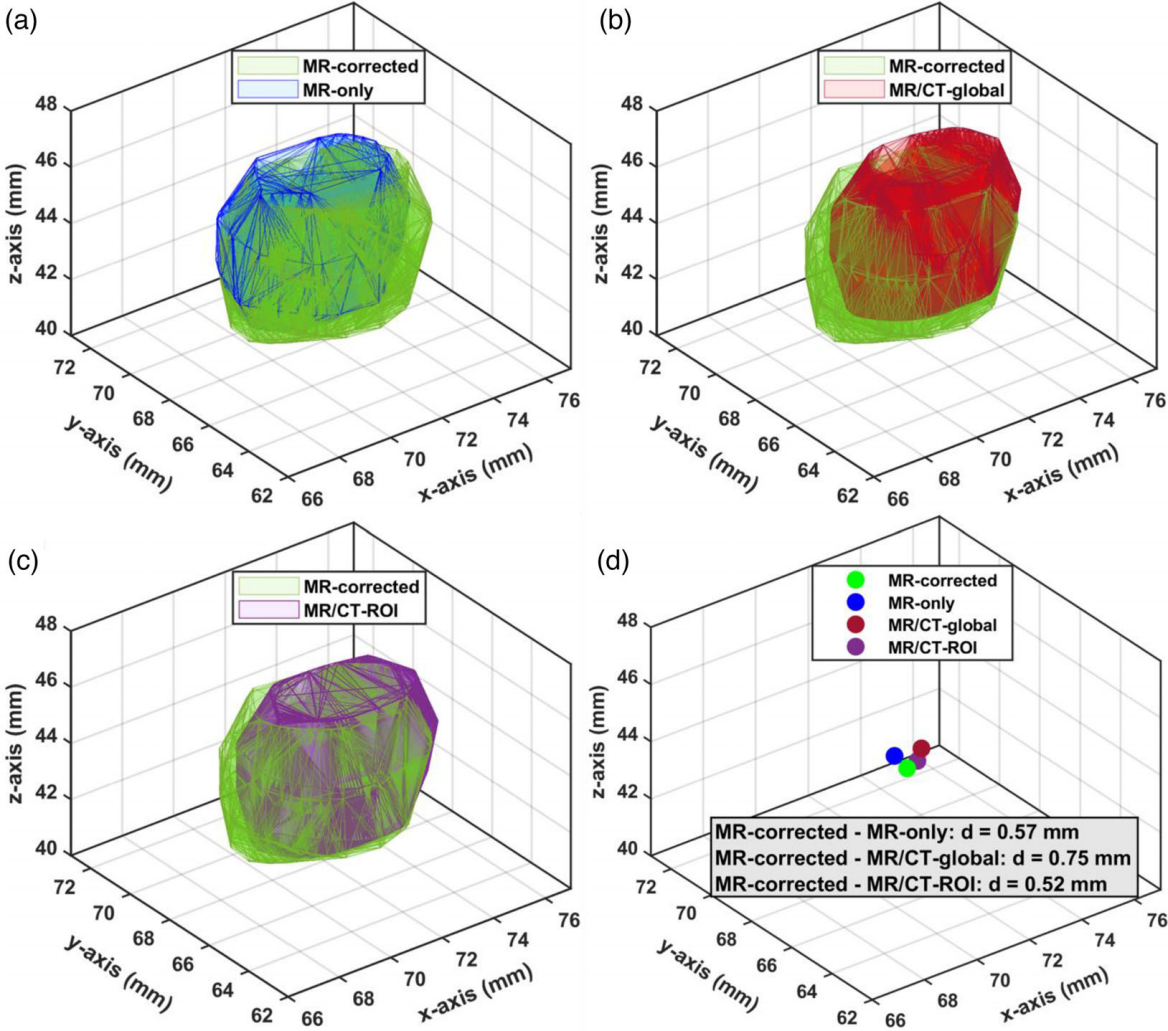
(A,B,C) An indicative brain lesion, contoured on the contrast‐enhanced T1‐weighted magnetic resonance (T1w MR) images and localized in the Leksell stereotactic space (LSS), following all four frame‐based workflows. Transformed structures are shown in 3D, in pairs of two for visibility and comparison purposes. (D) The position of the centroid corresponding to each workflow, localized in the LSS coordinate system and corresponding radial distances $d=\sqrt{\Delta x^{2}+\Delta y^{2}+\Delta z^{2}}$. *x*‐, *y*‐, *z*‐axes correspond to the normal axes of the LSS

In Figure 2, a selected indicative lesion (volume of 97.6 mm^3^, located at 69 mm from the MR isocenter) delineated on the clinical MR images and transformed to the LSS, following all considered workflows is shown, while in Table 4 results related to all 81 lesions are summarized. Although the same MR images were initially employed for lesion delineation, the spatial offset between transformed structures in the different workflows (using MR‐corrected workflow to define the reference position) is different (Figure 2).

**TABLE 4:** Summary of the patient study results, involving all 81 lesions. Median and maximum offset between structure centroids localized in the Leksell stereotactic space (LSS), with results related to the MR‐corrected workflow serving as the reference dataset for the evaluation of the clinical workflows

	Median absolute offset (mm)				Maximum absolute offset (mm)			
	Δ*x*	Δ*y*	Δ*z*	*R*	Δ*x*	Δ*y*	Δ*z*	*R*
MR‐only	0.1	0.3	0.4	0.6	0.4	0.9	1.0	1.1
MR/CT‐global	0.3	0.2	0.3	0.7	0.9	1.4	1.2	1.4
MR/CT‐ROI	0.2	0.2	0.2	0.4	0.9	0.9	0.9	1.1

*Note*: x, y, z correspond to the normal axes of the Leksell stereotactic space (LSS); *x*: left‐right direction; *y*: posterior‐anterior direction; *z*: inferior‐superior direction; *R*: radial distance between reference and evaluated points, i.e., $R=\sqrt{\Delta x^{2}+\Delta y^{2}+\Delta z^{2}}$.

Abbreviations: CT, computed tomography; MR, magnetic resonance.

Table 4 reveals that the MR/CT‐ROI workflow performs best in terms of median detected offset. Median offset values well below 1.0 mm have been observed for all workflows considered. Selecting a confined ROI around the central brain area instead of performing a global co‐registration step considerably improves TLA in MR/CT workflows.

**FIGURE 3:**
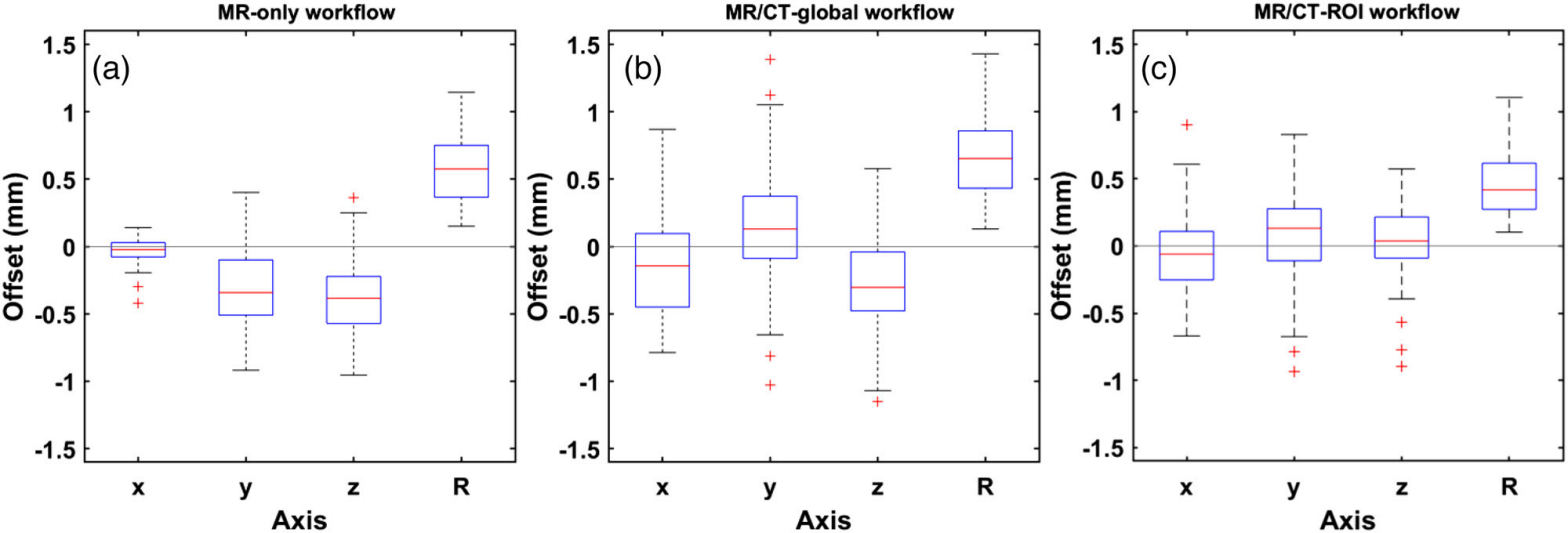
Box‐whisker plots derived from the patient study results, implementing the (A) magnetic resonance (MR) only, (B) magnetic resonance/computed tomography (MR/CT) global, and (C) MR/CT‐region of interest (ROI) workflows. Red lines indicate median detected offsets on each axis, whereas blue boxes range from the first to third quartile. Whiskers depict the remaining data or extend up to 1.5 times the interquartile range in either direction. In each dataset, remaining outliers (if any) are shown by the red marks. Legend: *x, y, z* correspond to the normal axes of the Leksell stereotactic space (LSS); *x*, left‐right direction; *y*, posterior‐anterior direction; *z*, inferior‐superior direction; *R*, the radial distance between reference and evaluated points, i.e., $R=\sqrt{\Delta x^{2}+\Delta y^{2}+\Delta z^{2}}$

To highlight outliers and quantitatively depict the distribution of TLA on each axis, Figure 3 presents relevant box‐whisker plots. With respect to the MR‐only workflow (Figure 3A), offsets detected on *x*‐axis are minimum. Corresponding values on *y*‐ and *z*‐axes are more scattered with a preferable negative sign for the vast majority of lesions, suggesting a systematic directionality of the evaluated dataset toward the negative *y*‐ and *z*‐axes. This can be attributed to the directionality of sequence‐dependent MR distortions, exhibited only on *y*‐axis, while *z*‐axis localization is indirectly affected by in‐plane displacement of the N‐shaped fiducials. This is not the case for both MR/CT workflows (Figure 3B,C), as results are scattered around the zero offset for all three axes, with no apparent systematic directionality.

**FIGURE 4:**
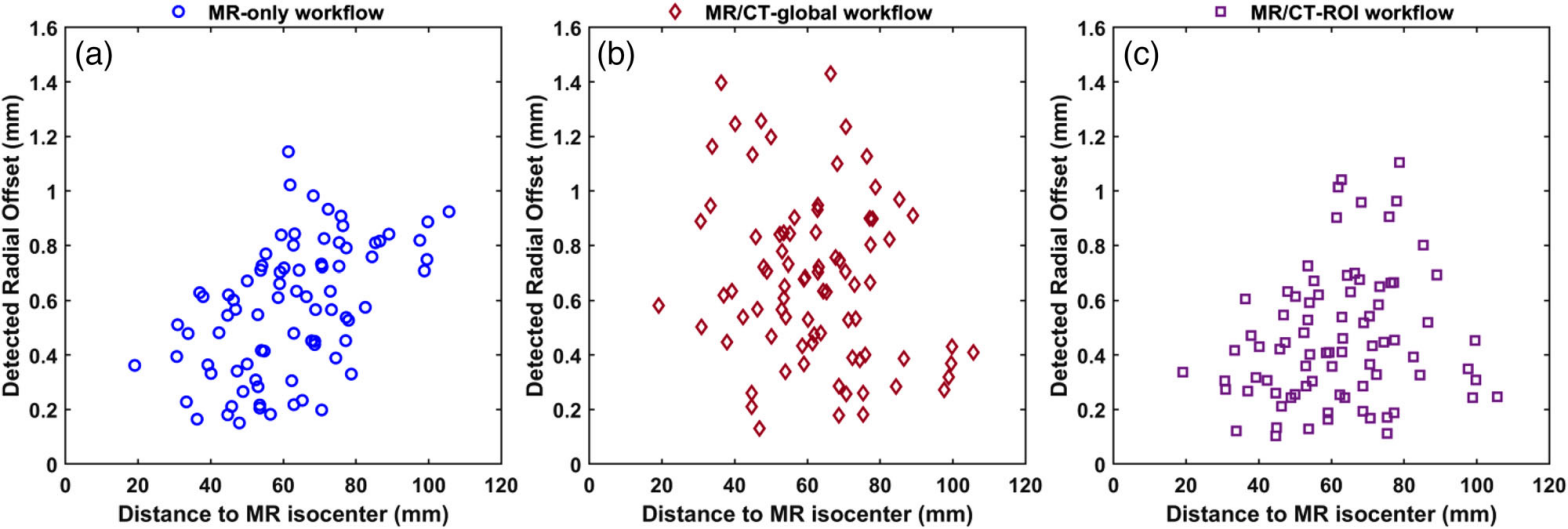
Detected radial offset between reference and evaluated centroid locations in the Leksell stereotactic space (LSS), plotted against distance to the magnetic resonance (MR) isocenter, for each lesion. (A) MR‐only workflow, (B) magnetic resonance/computed tomography (MR/CT)‐global workflow, (C) MR/CT‐region of interest (ROI) workflow. Using the Spearman's correlation coefficient, a statistically significant positive correlation was revealed only for the MR‐only workflow (see Section 3.2)

Figure 4 investigates the potential correlation of detected spatial offset with the structure's radial distance to the MR isocenter. Although all datasets are heavily scattered, for the MR‐only workflow, lesions lying distal to the MR isocenter tend to exhibit increased spatial offset (Figure 4). This is verified by calculating the Spearman's correlation coefficient. Specifically, a statistically significant moderate positive correlation between distance to MR isocenter and detected radial offset was revealed (Spearman's rho = 0.52, *p* < 0.001). On the other hand, for both MR/CT workflows the same analysis did not yield a statistically significant result at the 95% confidence level (*p* > 0.05), that is, there is not enough evidence to reject the null hypothesis that there is no correlation between distance to MR isocenter and detected spatial offset.

## 4 DISCUSSION

In volumetric imaging, spatial fidelity assessment is commonly performed by employing phantoms exhibiting adequate contrast in CT and MR images and involving distinct physical structures that serve as control points for geometric accuracy evaluation.^16^, ^17^, ^25^, ^31^, ^36^, ^37^, ^38^, ^39^, ^40^ The distribution of reference control points is often derived from the CT images (serving as the golden standard for spatial accuracy) or from mechanically predetermined positions. Distortion maps over a large volume of interest are deduced. Other studies have separately dealt with MR/CT co‐registration accuracy, using phantoms or patient images.^27^, ^28^, ^29^, ^30^, ^31^ In any case, the overall TLA, including MR distortion and its impact on MR/LSS or MR/CT/LSS spatial co‐registration accuracy, has not been explicitly determined. Calusi et al. recently reported in‐phantom targeting accuracy involving MR distortion and image co‐registration steps.^31^ However, in a phantom study patient‐induced MR distortions are not relevant, while MR/CT co‐registration is less prone to errors. In this work, clinical GK SRS workflows were evaluated in both phantom and patient studies, following the standard procedures for target determination and transformation to the LSS. The main advantage of this work is that spatial accuracy was determined specifically at the target locations, that is, at the high‐dose areas where strict geometric tolerances apply. This was facilitated by regarding the contoured targets, defined in the contrast (GD‐DTPA)‐enhanced T1w MR images and thus including any considerable Gd‐DTPA‐induced distortion,^24^ as the actual control points for spatial accuracy evaluation.

Phantom selection was challenging on the grounds of reference locations determination. A spherical container filled with 3D polymer gel dosimeter was irradiated using the GK treatment delivery unit after prescribing 26 4 mm shots directly in the LSS. As a result, signal reduction was induced to the T2w images at the high‐dose areas, offering the unique characteristic of introducing contrast/targets at well‐defined LSS coordinates. A similar approach has been followed (using either T1w or T2w images) in previous studies,^22^, ^23^ although none of them involved CT images and corresponding MR/CT co‐registration steps. Another advantage of the employed phantom is that it can be constructed in‐house, involving low‐cost materials. Experience in polymer gel dosimetry is not a prerequisite as dose measurements are not relevant in this methodology. The main drawback of the selected phantom is that it is spherical and homogeneous and, therefore, the MR/CT co‐registration step is less realistic, as compared to employing an anthropomorphic phantom instead.^31^, ^41^

A validation test was performed and uncertainty associated with the centroid localization algorithm was estimated at 0.16 mm (adopting the worst deviation detected). Regarding the phantom study, additional uncertainties related to the spatial accuracy of the treatment delivery unit should be considered. Thus, specific quality control checks were performed according to the recommendations described in Petti et al.^15^ An uncertainty of 0.1 mm was related to the mechanical accuracy of the robotic treatment couch and an additional uncertainty of 0.1 mm was associated with the coincidence of the RFP with the UCP. Summing the above three sources of uncertainty, a combined uncertainty of 0.21 mm is associated with the presented phantom study results. This is comparable to the median value of TLA of the MR‐corrected workflow, presented in Section 3.2. On the other hand, this uncertainty level does not directly apply to the patient study results as no dose delivery was performed. However, in the patient study, each offset determination required the calculation of two structure centroids (with the one defined using the MR‐corrected workflow serving as reference), that is, a combined uncertainty of 0.23 mm. Moreover, an extra 0.22 mm should be considered, associated with the uncertainty of reference centroid locations (reflected by the median offset of the MR‐corrected workflow determined in the phantom study, but used as reference in the patient study). Consequently, a total combined uncertainty of 0.32 mm should be ascribed to all patient study results (Section 3.3).

The phantom study was designed to allow for the evaluation of the TLA following all three workflows considered. Regarding the clinical ones, the MR/CT workflow performed slightly better than the MR‐only approach (Table 3). However, in a homogeneous phantom MR and CT image signal, contrast, and sharpness are higher, without exhibiting considerable noise, blurring, or partial volume effects between structures. Thus, it can be expected that under optimum image quality, the MR/CT co‐registration algorithm performs better compared to patient images. In the recent work of Calusi et al., in‐phantom targeting accuracies of 0.8 ± 0.9 mm and 1.0 ± 0.9 mm were reported for the MR‐only and MR/CT workflows, respectively, evaluated at a single point using ion chamber measurements.^31^ These values are in agreement with the corresponding ones reported herein, but are not necessarily relevant in a patient study.

For 15 cases of multiple brain metastases, involving a total of 81 lesions, the clinical frame‐based workflows were evaluated. Median offsets on all axes were in good agreement with the corresponding ones obtained using the phantom. However, the maximum values related to the MR/CT‐global workflow were slightly higher than phantom study results (Figure 3; Tables 3 and 4). This can be attributed to the larger number of targets considered in the patient study (81 as compared to 26) and, most importantly, the real clinical image characteristics and quality (e.g., noise, contrast, sharpness, etc.) that could have affected the accuracy of the MR/CT spatial co‐registration step. A ROI‐based co‐registration improves the TLA as reflected in both median and maximum offsets detected for the MR/CT‐ROI workflow (Table 4). Further limiting the volume of interest used for image co‐registration around each lesion could potentially improve the overall spatial accuracy. However, since CT images were not contrast‐enhanced, a sub‐optimum image fusion might go undetected due to lack of reference anatomical landmarks within the volume of interest (such as contrast‐enhanced vessels or normal organs as described in Brock et al.^29^). Nevertheless, the most optimum image fusion is also user‐dependent and partly subjective. The AAPM TG‐132 recommends that for stereotactic use, the entire image volume is aligned to within 1 mm which corresponds to level zero in the uncertainty registration scale.^29^ Chung et al. investigated the registration error after using three smaller brain subregions (ROI‐based co‐registration), as well as using the entire skull volume as the volumes of interest (global co‐registration).^30^ Results were compared with fiducial‐based registration. According to that study, a ROI‐based co‐registration similar to that used in the present study resulted in slightly decreased registration uncertainty, as compared to the entire skull volume.

Several concerns related to this study might limit the applicability of the presented results. First of all, MR distortion magnitude greatly varies with several MR scanning parameters, such as sequence type, echo time, shimming, receiver bandwidth, contrast agent uptake, lesion proximity to the base of the headframe, and others.^16^, ^17^, ^24^, ^26^, ^36^ In this work, the clinical pulse sequences and imaging parameters were used. However, other institutions employing scanners of different specifications might commission an SRS workflow employing considerably different imaging parameters. In such case, it is expected that both MR‐only and MR/CT TLA are affected. Furthermore, 3.0T MR imaging was not studied, although respective scanners are known to exhibit increased sequence‐dependent distortion.^3^, ^16^, ^42^, ^43^, ^44^ However, it should be noted that this effect can be counterbalanced by increasing the receiver bandwidth. Spatial resolution is another parameter not investigated for its impact on the overall TLA. As an example, in a recent study, MR slice thickness selection was associated with the number of brain metastases identified, as well as the target volume contoured for SRS treatment planning.^45^ In addition, the effect of further varying the volume of interest used for anatomy‐based MR/CT co‐registration in the overall TLA was not explicitly investigated. Moreover, several brain metastases were excluded from the patient study of this work due to presence of necrotic cores or being adjacent to vessels (see selection criteria in Section 2.2.1). The methodology followed herein would not be applicable to these lesions. However, such cases are more prone to susceptibility‐related distortions and, therefore, require more specific investigation. Finally, patient's MR images were obtained with the headframe on, thus avoiding movement‐related artifacts that could affect TLA in MR/CT workflows. However, in clinical practice MR imaging is commonly performed without the headframe. Movement artifacts in MR imaging degrade image quality and may lead to misinterpretation especially for small targets introducing displacements of the order of 1 mm for brain imaging.^46^ Thus, results presented in this study do not directly apply in thermoplastic mask‐based workflows.

Discussion on application of margins for target volume definition in SRS for the management of multiple brain metastases is a long‐lasting debate, involving zero‐ or sub‐millimeter margin approaches and reaching up to 3 mm.^10^, ^11^, ^12^, ^47^, ^48^ Other studies, focusing on linac‐based SRS, have tried to associate lesion location or volume with the selection of a suitable margin.^3^, ^4^, ^8^ Overall results of this work could be useful in the determination of a margin strategy followed specifically in GK frame‐based SRS applications, as well as in applications with other systems following MR/CT co‐registration approaches. According to the obtained results, in an MR‐only workflow, spatial accuracy related to target localization in the LSS can be estimated at 0.6 mm (median offset) and slightly exceeded 1.0 mm for a few targets. The MR/CT‐ROI workflow exhibited improved TLA (median offset of 0.4 mm) as compared to the MR/CT‐global workflow but maximum detected offsets also exceeded 1 mm. Nevertheless, a ROI‐based MR/CT co‐registration approach should be preferred instead of a global registration one for MR/CT workflows. Considering the results of this study as indicative for GK frame‐based treatment workflows and taking into account the limitations discussed, a subpixel TLA is achievable, but cannot be guaranteed for all cases. However, margin definition is more complex as mechanical and dose delivery uncertainties need to be taken into account as well.^15^ In addition, interobserver variability in target delineation might also be the most dominant contributor, especially for cases involving very small lesions.^35^

## 5 CONCLUSIONS

The developed methodology is suitable for detecting target displacements in the coordinate system of the LSS associated with MR distortion at the lesion locations and/or sub‐optimum spatial co‐registration. Summing all sources of uncertainty involved, a combined uncertainty of 0.21 and 0.32 mm should be ascribed to the phantom and patient study results, respectively.

Overall results of this work suggest that a subpixel TLA is achievable in GK frame‐based workflows, but cannot be guaranteed for all cases. The accuracy was found to depend on the workflow followed. For the MR/CT workflow, a ROI‐based MR/CT co‐registration approach could considerably increase TLA and should be preferred instead of a global registration.

Presented methods could be implemented for the determination of a site‐, modality‐, and workflow‐specific margin strategy in SRS treatment planning.

## CONFLICT OF INTEREST

The authors have no conflict of interest to disclose.

## AUTHOR CONTRIBUTIONS

Study conception and design: Pantelis Karaiskos. MR sequence selection and optimization: Ioannis Seimenis. Contour review and verification: Stefanos Lampropoulos and Georgios Kollias. MR/CT co‐registration review and verification: Stefanos Lampropoulos and Georgios Kollias. Development of image processing routines: Eleftherios P. Pappas, Panagiotis Kouris, and Stefanos Theocharis. Data analysis: Eleftherios P. Pappas and Panagiotis Kouris. Data interpretation: Pantelis Karaiskos, Ioannis Seimenis, and Eleftherios P. Pappas. Preparation of figures and tables: Eleftherios P. Pappas. Literature review: Pantelis Karaiskos, Ioannis Seimenis, and Eleftherios P. Pappas. Manuscript preparation: Eleftherios P. Pappas, Pantelis Karaiskos, and Ioannis Seimenis. Manuscript editing: all authors.
